# Non-malarial infectious diseases of antenatal care in pregnant women in Franceville, Gabon

**DOI:** 10.1186/s12884-017-1362-0

**Published:** 2017-06-12

**Authors:** Irene Pegha Moukandja, Edgard Brice Ngoungou, Guy Joseph Lemamy, Ulrick Bisvigou, Antoine Gessain, Fousseyni S. Toure Ndouo, Mirdad Kazanji, Jean Bernard Lekana-Douki

**Affiliations:** 1 Unite de Parasitologie Medicale (UPARAM), Centre International de Recherches Medicales de Franceville (CIRMF), BP 769 Franceville, Gabon; 2 Departement de Parasitologie-Mycologie Medecine Tropicale, Faculte de Medecine, Universite des Sciences de la Sante, BP 4009 Libreville, Gabon; 3 Departement de Sante Publique et de Medecine Legale et du Travail, Faculte de Medecine, Universite des Sciences de la Sante, B P 4009 Libreville, Gabon; 4 Unite d’Epidemiologie et Physiopathologie des Virus Oncogenes, Institut Pasteur, Paris, France; 5 Institut Pasteur de Bangui, Bangui, République Centrafricaine; 6 Departement de Biologie Cellulaire et Moleculaire Universite des Sciences de la Sante, B P 4009 Libreville, Gabon; 7 Present address:UPARAM, CIRMF, B P 769 Franceville, Gabon

**Keywords:** Pregnant women, Antenatal care, Rubella virus, HIV, HTLV1, *Troponema pallidum*, *Toxoplasma gondii*, Gabon

## Abstract

**Background:**

In sub-tropical countries, infectious diseases remain one of the main causes of mortality. Because of their lack of active immunity, pregnant women and their unborn children represent the most susceptible people. In Gabon, data on infectious diseases of pregnant women such as syphilis and rubella are either scarce or very old. Few studies have assessed *T. gondii* infection during pregnancy in the country. Here, we evaluate seroprevalence of HIV, HTVL-1, syphilis and *T. gondii* and rubella infection during antenatal care among women living in Franceville, Gabon.

**Methods:**

A retrospective study was conducted on data collected from May 2007 to July 2010. After signing an informed written consent form, all pregnant women consulting in two hospitals of Franceville (Gabon) and in offices of maternity and childbirth health centers were included. Demographic and clinical data were collected. Serum samples were collected and analysed using immunological assays relevant for HIV (Genscreen HIV-1 version 2, Bio-Rad®, Marne la Roquette, France).HTLV-1 (Vironostika HTLV-1, Biomérieux®, Marcy l’Etoile, France), *T. pallidum* (TPHA/VDRL), BIOLABO®SA), rubella virus (Vidas Biomerieux®, Marcy l’Etoile, France) and *T. gondii* (Vidas Biomerieux®, Marcy l’Etoile, France) diagnoses were performed. Data analysis was done using the Stat view 5.0 software.

**Results:**

A total of 973 pregnant women were assessed. The mean age was 25.84 ± 6.9 years, with a minimum age of 14.0 years and a maximum of 45.0 years. Women from 26 to 45 years old and unemployed women were the most prevalent: 41.93% and 77.18%, respectively. The prevalence of studied infectious diseases were 2.50% for syphilis, 2.88% for HTLV-1, 4.00% for HIV with no significant difference between them (*p* = 0.1). Seropositivity against rubella was higher (87.56%, *n* = 852) than seropositivity against *T. gondii* (57.35%, *n* = 557), (*p* < 0.0001). Only 5 (0.51%) co-infection cases were found: 2 co-infected with HIVand *T. pallidum*, 2 co-infected with HIV and HTLV-1, and one co-infected with *T. pallidum* and HTLV-1. Sixty-two pregnant women were seronegative against toxoplasmosis and rubella (6.37%).

**Conclusion:**

High levels of seropositivity against *T. gondii* and the rubella virus were observed. The prevalence of *T. pallidum* and HTLV-1 were lowest but HIV prevalence in young women was worrying.

## Background

In recent years, health care has globally improved thanks to the introduction of a number of vaccines, the use of antibiotics and the development of antiretroviral medication for the treatment of HIV/AIDS. Unfortunately, infectious diseases remain a major cause of mortality in developing countries. More specifically, infections due to viruses, bacteria, fungi and parasites continue to create havoc and lead to a great morbidity which hinders development in resource-limited countries. These infectious agents do not induce systematically diseases, pathogenesisis also associated with host genetics, host immunity, infectious agent genetics and environmental factors.

Pregnancy is a specific physiological state in which the woman must tolerate the fetus, a foreign organism for the mother, since half of its genetic material is inherited from the father. Intuitively, the fetus should be rejected by the mother. However, the mother’s body naturally accepts the fetus by undergoing immune and morpho-physiological changes [1, 2]. Compared to non-pregnant women, the pregnant mother’s immune system is more susceptible to infectious diseases [3, 4]. Several consequences of infections during pregnancy have been associated with severe complications affecting the mother and/or the fetus and some health problems even lead to the death of one, the other or both [5]. Among these infections, HIV, *Treponema pallidum*, *T. gondii*, the rubella virus and human T-cell lymphotropic virus type 1 need a rigorous monitoring.

The World Health Organization(WHO) estimates that 1.4million pregnant women live with HIV in developing countries and that 90% of these women live in Africa [6]. In pregnant women, HIV infection is responsible for several poor health outcomes such as premature birth, low birth weight, miscarriage or newborn mortality [7–10].Although in utero transmission of HIV rarely occurs, during delivery, 65% of mothers transmit the virus to their child [11].

Syphilis is caused by *Treponema pallidum* spirochete bacteria and the transmission risk from mother to fetus during pregnancy is quite high. This phenomenon was first described in 1497 [12, 13]. The main risk factors of *T. pallidum* transmission from mother to child are the evolution of maternal infection and in utero exposure time [10, 14]. Recent systematic reviews and meta-analysis show that syphilis is responsible for an increased frequency of fetus loss and stillbirth (21%), neonatal deaths (9.3%) and low birth weight (5.8%), when it is untreated in pregnant women [15]. Clinical evidence of congenital syphilis was seen in 15% of newborns from untreated women and the frequency of the disease was 10% higher than in babies from treated mothers [15]. *T. pallidum* transmission to the child can occur by blood diffusion or during the delivery by direct contact with the mother’s genital lesions.

Human T-cell lymphotropic virus type 1 (HTLV-1) is a human retrovirus causing adult T-cell leukemia/lymphoma (ATL) and tropical spastic paraparesis/HTLV-1-associated myelopathy (TSP/HAM) [16, 17].HTLV-1 has also been associated with several inflammatory diseases, including pediatric infectious dermatitis [18, 19], uveitis [20, 21], and myositis [19, 22]. About 15 to 20 million infected people live with HTLV1 in endemic areas which include Japan, parts of sub-Saharan Africa, the Caribbean, South-America and the Middle-East regions [23]. Globally, HTLV-1 is prevalent in more than 2% of the adult population, and 2 to 8% of these infected individuals will develop severe HTLV-1-associated diseases such as adult T-cell leukemia/lymphoma and tropical spastic paraparesis/HTLV-1-associated myelopathy during their lifetime [24].

Toxoplasmosis is a parasitic disease caused by the protozoan *Toxoplasma gondii.* In pregnant women, although 29% of congenital *T. gondii* infections are usually asymptomatic, the infection can be delayed or become potentially severe once it manifests [25].Transmission of *T. gondii* from mother to the fetus depends on parasitaemia, the mother’s cellular immunity as well as the stage of placental development [26]. Congenital toxoplasmosis can lead to several symptoms including mild chorioretinitis, an inflammation of the choroid (a thin pigmented vascular coat) and the eye retina, which can persist in the child many years after birth and can cause miscarriage, intellectual disability, micro cephaly, hydrocephalus and seizures [25].

Rubella, also known as German measles, is an infection caused by the rubella virus. In pregnancy, this infection poses a high risk for abnormal fetal formation when it occurs in the first 11 weeks of gestation. This risk seems to be nonexistent after the18th week of pregnancy [27]. Rubella in pregnant women has been associated with spontaneous abortion and congenital abnormalities such as cataracts, heart defects and sensorineural deafness. Permanent abnormalities such as heart defects, pulmonary artery stenosis, hypoplasia, eye defects, intellectual and psychomotor disabilities, and speech defects have also been linked to rubella infection. Other outcomes of rubella during pregnancy include transient abnormalities in newborns and infants, developmental and late-onset abnormalities [28, 29]. Retardation of intrauterine growth seems to be the only consequence of infection in the third trimester of pregnancy [27].

In Gabon, infectious diseases are more prevalent than in other geographical zones of the world [30, 31]. Although data on malaria in Gabonese pregnant women are available, current reports on other infectious diseases that threaten the health of the mother, fetus and the newborn children are rare and scattered. Data on syphilis in pregnancy are quite old [32] and, although some research has been performed on toxoplasmosis, few of these studies have focused on pregnant women [33, 34]. As for rubella, it is rare to find data on this disease in Gabonese pregnant women. There is a dire need for new studies and new data on the infectious diseases of pregnant women in Gabon in order to assess the health risks for mothers and their children.

**Fig. 1:**
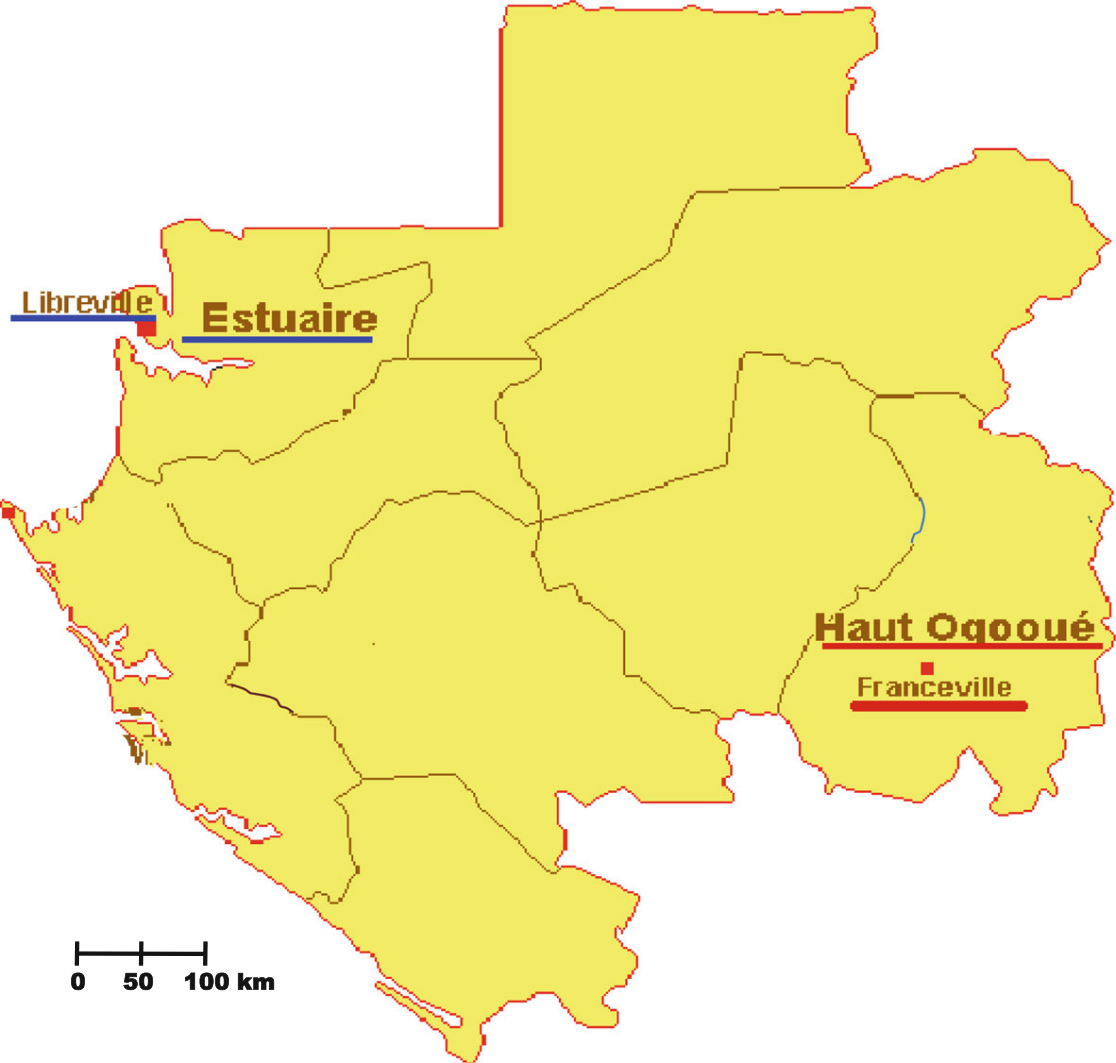
Map of Gabon. Libreville: capital of Gabon. Franceville: capital of province of Haut-Ogooué by Irène Pegha Moukandja

Franceville is the capital of the Haut-Ogooué province in the south-east of Gabon. It is located 512 km south-east of the country’s capital, Libreville (Fig. 1). Prevalences of HIV and HTLV in this city were reported in previous works [18, 35]. As is the case in the rest of the country, antenatal visits in pregnancy are concerned with HIV, rubella, syphilis, toxoplasmosis, malaria and hepatitis Band C. Therefore, the aim of this study was to evaluate the prevalence of these non-malaria infectious diseases diagnosed during the prenatal assessment of pregnant women in Franceville from May 2007 to July 2010.

## Methods

### Population and study sites

The following Franceville health facilities were included: a Mother and Child Health office (In French, *Santé Maternelle et Infantile*, SMI), two reference hospitals (the *Centre Hospitalier Régional “AmissaBongo” de Franceville*, CHRABF, and the “*Hôpital de l’Amitié Sino-Gabonaise*, HASG), as well as a private health center (Ménaye) in Franceville. We included pregnant women after obtaining their written informed consent and performing the antenatal care at the study sites. This retrospective study was conducted on data collected during a cross-sectional study of HTLV-1that occurred from May 2007 to July 2010.

Age and social status (i.e. worker, student, unemployed) were collected as demographic data.

### Diagnosis

For all the tests employed, only IgG were identified.

HIV was diagnosed by ELISA tests (Genscreen HIV-1/2 version 2, Biorad®, Marne la Coquette, France) and rapid tests (HIV-1/2 Determine™, Abbott, Chicago, USA).*T. pallidum* was diagnosed using serological tests (*Treponema pallidum* Hemagglutination Assay [36]/Venereal Disease Research Laboratory (VDRL), BIOLABO® South Africa). *T.pallidum* quantification was assessed using Phosphorothionate, 2-butenoic acid-3-(diethoxyphosphinothioyl) methyl ester (RPR-II) nosticon flocculation tests.

Women who were positive for HIV and *T. pallidum* were referred for treatment according to the national health policies in Gabon.

The serological diagnosis of rubella was conducted with the use of the ELFA test (Vidas Biomerieux®, Marcy l’Etoile, France). Samples were considered positive when dilution was ≥10 IU/ml. *T. gondii* infection was diagnosed using the VIDAS serological test (BioMérieux®, Marcy l’Etoile, France), IgG-avidity tests and the fluorescent enzyme-linked assay (ELFA) technique. When a woman had antibodies ≥10 IU/ml, against rubella and/or toxoplasmosis, she was considered seropositive against these infections.

Pregnant women who were positive for rubella and *T. gondii* had antibodies against these infectious agents suggesting previous infections or acquired immunity. This immune status was considered as protection.

### Statistical analysis

Statistical analysis was performed with the use of Stat view 5.0 (SAS Institute, Cary, USA). A chi-square test was used to compare quantitative variables among groups. The nonparametric Mann– Whitney U test, Pearson’s test and Fisher’s exact test were used for group comparisons, as appropriate. *p* values <0.05 were considered as statistical significance.

### Ethical aspects

All the pregnant women underwent a written informed consent process before enrolling in the study. This study was approved by the ethic committee of Gabonese Health Ministry (MSP/MD/134/2008).

## Results

### Distribution of sexually transmitted infectious diseases by age groups and social strata

A total of 973 pregnant women were included in the study. The mean age was years, with a range of 14.0-45.0 years. Women aged from 26 to 45 years old (41.93%) and unemployed women (77.18%) were the most prevalent, (Table 1). The prevalence of syphilis (2.50%), HTLV-1 (2.88%), and HIV (4.00%) were not significantly different (*p* = 0.1). Furthermore, the prevalence of HIV in women aged 26-45 years old (5.64%, *n* = 23) was higher than in women aged 19-25 years old (3.02 (2.86-3.18)) (*p* = 0.05). There was no significant difference in HIV prevalence according to social class (*p* > 0.4). The prevalence of HTLV-1 was significantly higher in women aged 26-45 years old than in the 14-18 years old (*p* = 0.04) age group. However, there was no difference in HTLV-1 prevalence among the 14-18 years old, 18-25 years old, and 26-45 years old age groups. Social status did not influence the prevalence of HTLV-1 (*p* > 0.1). For *T. pallidum*, there was no significant difference in the prevalence either according to age or the social status (*p* > 0.1).

**Table 1:** Distribution of sexually transmitted infectious diseases by age groups and social strata

Age-group (years) n %, (95 CI)					Social strata n %, (95 CI)			
	[14-18] *n* = 265	[19-25] *n* = 300	[26-45] *n* = 408	*p* value	Worker *n* = 79	Student *n* = 143	Unemployed *n* = 751	*p* value
HIV +	8	8	23		3	4	32	
*n* = 39/973 4.01% [3.79-4.23]	3.02 (2.86-3.18)	2.67 (2.54-2.80)	5.64(5.32-3.96)	*P* = 0.09	3.80(2.60-4.01)	2.80 (2.65-2.94)	4.26 (4.03-4.49)	*P* = 0.71
HTLV-1 +	3	10	15		2	5	21	
*n* = 28/973 2.88% [2.73-3.03]	1.13 (1.10-1.15)	3.33 (3.16-3.50)	3.68 (3.48-3.88)	*P* = 0.13	2.53 (2.41-2.65)	3.50 (3.31-3.69)	2.80 (2.66-2.94)	*P* = 0.88
*T. pallidum* +	7	4	13		3	1	20	
*n* = 24/973 2.5% [2.38-2.62]	2.64 (2.51-2.77)	1.33 (1.29-1.37)	3.19 (3.02-3.36)	*P* = 0.29	3.80(2.60-4.01)	0.70 (0.67-0.72)	2.66 (2.53-2.79)	*P* = 0.29

### Seroprevalence of the rubella virus and *T. gondii* by age groups and social status

The prevalence of pregnant women who were seronegative against *T. gondii* (42.75%, *n* = 416) was higher than those seronegative against rubella (12.44%, *n* = 121), (*p* = 7.4.10^−30^), (Table 2). The prevalence of pregnant women who were seronegative against *T. gondii* decreased as age increased (48.68%, 44.00%, 37.99% for the 14-18, 18-25 and 25-45 age groups respectively, *p* = 0.02). However, the prevalence of these diseases did not vary according to social status (*p* > 0.09). Similarly, the prevalence of seronegative women against rubella decreased asage increased (21.51%, 11.33%, 7.35% for the 14-18, 18-25 and 26-45 age groups respectively, *p* < 0.002), (Table 3). Furthermore, the prevalence of seronegativity against rubella in the student group (19.58%, *n* = 28/143) was higher than in the worker (7.59%, *n* = 6/79) and in the unemployed group (11.58, *n* = 87/751, *p* < 0.01), (Table 4).

**Table 2:** Seronegativity of the rubella virus and *T. gondii* by age groups and social strata

	Age-group (years) n %, (95 CI)				Social strata n %, (95 CI)			
	[13–17] 265	[18–24] 300	[25–44] 408	*p* value	Worker 79	Student 143	Unemployed 751	*p* value
*T. gondii* 416/973 42.75% [40.10-45.40]	129 48.68 (45.65-51.71)	132 44.00 (41.26-46.74)	155 37.99 (35.64-40.34)	*P* = 0.02	27 34.18 (32.06-36.30)	60 41.96 (39.36-44.57)	329 43.81 (41.08-46.53)	*P* = 0.25
Rubella 121/973 12.44% [11.68-13.20]	57 21.51 (20.49-22.82)	34 11.33 (10.65-12.01)	30 7.35 (6.91-7.79)	*P* < 0.002	6 7.59 (7.15-8.03)	28 19.58 (18.39-20.77)	87 11.58 (10.88-12.60)	*P* < 0.012

**Table 3:** Distribution of seronegativities of *T. gondii* and rubella among pregnant women infected by sexually transmitted diseases

	*T. pallidum* infected women n % [95%IC]	HTLV1 infected women n % [95%IC]	HIV infected women n % [95%IC]	rubella seronegatives women n % [95%IC]	*T. gondii* seronegatives women n % [95%IC]
*T. gondii* seronegatives women	9 0.92% [0.90-0.94]	15 1.54%[1.48-1.60]	12 1.23% [1.20-1.26]	62 6.37% [6-6.74]	
Rubella seronegatives women	1 0.10% [0.08-0.14]	3 0.31% [0.29-0.33]	6 0.62% [0.59-0.65]		
HIV infected women	2 0.21% [0.18-0.24]	2 0.21% [0.18-0.24]			
HTLV1 infected women	1 0.10% [0.08-0.14]				
*T. pallidum* infected women

**Table 4:** Relation between HIV and the other four infections

	HIV+ (n)	OR (95% CI)	*p* value
*T. pallidum*			0.274
Negative	22	1	
Positive	2 2.24	(0.24-9.69)	
HTLV1			0.390
Negative	37	1	
Positive	2	1.89 (0.21-8.03)	
Rubella virus			0.575
Negative	12	1	
Positive	27	1.72 (0.83-3.76)	
*T. gondii*			0.123
Negative	6	1	
Positive	33	0.77 (0.31-2.31)	

There was a significant difference between HIV prevalence and seronegative *T. gondii* prevalence (*p* = 5.37.10^−91^). The disease prevalence was also significantly different for syphilis and rubella-seronegative women (*p* = 3.78.10^−15^).

### Co-infections of sexually transmitted diseases in seronegative women with *T. gondii*

Some co-infection cases were found (Table 5 and Table 6) which included two (2; 0.20%) women with HIV and *T. pallidum* infections. Two (2; 0.20%) other women were infected with both HIV and HTLV-1, whereas one (1; 0.2%) woman with *T. pallidum* was infected by HTLV-1. There were also pregnant women who were neither seropositive against *T.gondii* nor against rubella (*n* = 62; 6.37%). Twelve (1.23%) of the women infected with HIV were seronegative against *T.gondii*. We also found six (0.62%) HIV-infected women who were seronegative against rubella while 15 (1.54%) women infected with HTLV-1 were also seronegative against *T.gondii*. Among the women who were seronegative against *T. gondii* and seronegative against the rubella virus, two (0.20%) were infected by HTLV-1; one was unemployed and the other was a worker. Also, two (0.20%) women who were seronegative against both *T. gondii* and the rubella virus were HIV infected (one unemployed and one student). There were no *T. pallidum* infections in women seronegative against *T. gondii* or against rubella. None of the women were simultaneously infected with all three infectious agents: HIV, HTLV-1 and *T. pallidum*.

**Table 5:** The associations between HIV and others infections stratified by age

	HIV + (%)	OR (95% CI)
Stratified by age		
Syphilis (n = 2)		
[14-18] (n = 0)	0	1
[19-25] (n = 1)	50.0	6.12 (0.12-62.7)
[26-45] (n = 1)	50.0	1.47 (0.03-10.78)
Chi2: 0.305 Crude OR brut (95% CI): 2.24 (0.25-9.69) Adjusted OR (95% CI): 2.13 (0.48-9.44)		
HTLV1(n = 2)		
[14-18] (n = 0)	0	1
[19-25] (n = 1)	50.0	3.01 (0.63-25.23)
[26-45] (n = 1)	50.0	1.25 (0.03-8.97)
Chi2: 0.469 Crude OR brut (95% CI): 1.89 (0.21-8.03) Adjusted OR (95% CI): 1.71 (0.39-7.53)		
Rubella (n = 33)		
[14-18] (n = 4)	12.12	1
[19-25] (n = 8)	24.24	0.34 (0.08-2.08)
[26-45] (n = 21)	63.64	0.54 (0.15-3.01)
Chi2: 0.293 Crude OR (95% CI): 0.78 (0.31-2.31) Adjusted OR (95% CI): 0.63 (0.26-1.54)		
Toxoplasmosis (n = 2)		
[14-18] (n = 2)	7.41	1
[19-25] (n = 6)	22.22	0.93 (0.23-3.95)
[26-45] (n = 19)	70.37	
Chi2: 0.169 Crude OR (95% CI): 1.72 (0.83-3.76) Adjusted OR (95% CI): 1.63 (0.81-3.28)

**Table 6:** Analyze of the associations between HIV and others infections stratified by the socioeconomic status

	HIV + (%)	OR (95% CI)
*Stratified by occupation*		
*Syphilis (n = 2)*		
*Student* *(n = 0)*	00.0	1
*Unemployed* *(n = 2)*	100.0	2.60 (0.28-11.66)
*Worker* *(n = 0)*	00.0	
*Chi2*: *0.297* *Crude OR brut (95% CI)*: *2.24 (0.25-9.69)* *Adjusted OR (95% CI): 2.17 (0.49-9.60)*		
*HTLV1 (n = 2)*		
*Student* *(n = 0)*	00.0	1
*Unemployed* *(n = 2)*	100.0	2.46 (0.27-10.96)
*Worker* *(n = 0)*	00.0	
*Chi2*: *0.760* *Crude OR brut (95% CI)*: *1.89 (0.21-8.03)* *Adjusted OR (95% CI): 1.91 (0.44-8.38)*		
*Rubella (n = 33)*		
*Student* *(n = 3)*	9.09	1
*Unemployed* *(n = 28)*	84.85	0.92 (0.31-3.69)
*Worker* *(n = 2)*	6.06	0.14 (0.01-9.96)
*Chi2*: *0.525* *Crude OR (95% CI)*: *0.78 (0.31-2.31)* *Adjusted OR (95% CI): 0.75 (0.32-1.83)*		
*Toxoplasmosis (n = 27)*		
*Student* *(n = 2)*	7.41	1
*Unemployed* *(n = 22)*	81.48	1.75 (0.78-4.21)
*Worker* *(n = 3)*	11.11	
*Chi2*: *0.119* *Crude OR (95% CI)*: *1.72 (0.83-3.76)* *Adjusted OR (95% CI): 1.73 (0.86-3.47)*		

## Discussion

Infectious diseases remain important risk factors for the health of mothers and their unborn children during pregnancy. This study is not only one of the few investigations of these conditions in Africa, it is also the first study in Gabon focusing on several non-malarial infections during pregnancy. The HIV prevalence in pregnant women found here is lower than the one previously reported in Gabon in studies on women in similar age groups (26 to 45 years old) [35], showing the regional specificity of HIV prevalence in the same country. This could be explained by socio-cultural factors. The reported HIV prevalence in this study has also increased compared to reports in similar studies conducted in Franceville since 1997 [34, 37, 38]. This could be one of the consequences of the decrease of means to fight against HIV these past few years in Gabon. Studies conducted in other African countries have shown similar HIV prevalence. A similar study conducted from 2009 to 2010 on Malian pregnant women found a comparable HIV prevalence (4.1%). In 2004, a study on rural Cameroonian pregnant women also reported similar HIV incidence (4.0%) [39, 40]. However, HIV prevalence reported in the Central African Republic in 2006 was higher (15%) [41], suggesting a difficulty to fight HIV in areas with political instability.

Age seemed to affect HIV prevalence in this study since a relatively higher HIV prevalence was observed in younger women. It suggests a high exposure and vulnerability of HIV infection among women in the younger age groups and potential risks of vertical transmission during their pregnancies in the future. Furthermore, HIV prevalence was also higher in our study compared to similar investigations from India and Europe [42, 43], although in those parts of the world, HIV pregnant women seem to be older (26 years and older) [42]. This could be related to socio-cultural habits.

In 2005, a study of 161 pregnant women in Franceville reported a 5% prevalence for HTLV-1 in this population [18] which is not significantly different from the 2.88% prevalence found in our study. It should be noted that the smaller sample size of the 2005 study may explain the observed difference in our findings. Our results, similar to those of a previous national HTLV-1 study conducted in 2005, confirm a decrease in HTLV-1 prevalence based on a 1986 Gabonese study [18, 34]. In studies conducted in Europe and in the United States of America (USA), the prevalence of this infection increased with age [44, 45] which is probably due to the accumulation of new infections through incremental sexual activity over a lifetime [46, 47]. We did not investigate genetic diversity for this virus, but it was previously found that HTLV-1b was the major subtype in Gabon and a few viral strain subtypes have also been found in pregnant Gabonese women [18].

The prevalence of *T. pallidum* reported in our study (2.5%) appears to be similar to prevalence reports from India [48, 49]. Surprisingly, in our study, age did not seem to significantly affect *T. pallidum* prevalence; the low prevalence of this infection could explain this finding. Our study shows a decline in the prevalence of *T. pallidum* when compared to the 10% prevalence found in Gabonese pregnant women in 1988 [50].This prevalence in our study population was lower than the one reported in the Democratic Republic of the Congo in 2011 (4.2%) and similar to the one reported in Benin (2.5%) [51, 52]. Syphilis is one of the infectious diseases that is of great interest to the global health community. The WHO recommends screening for *T. pallidum* in routine antenatal care visits in the first trimester of pregnancy in order to prevent adverse health pregnancy outcomes such as stillbirth, neonatal death, low birth weight and congenital deformities [52]. Data obtained from our studies provides empirical evidence to support the pursuit of the elimination of this disease. Syphilis is one of the diseases for which one of the health goals is eradication by using available antibiotherapies. Its residual prevalence indicates a resistance from some people to follow safe sexual behaviors. *T. pallidum* is a good indicator of sexual behaviors, which is confirmed by the fact that no significant difference was observed in the frequencies of sexually transmitted infections studied here. Moreover, we found that 3/24 (12.5%) pregnant women with syphilis were infected by one other sexually transmitted infection confirming that an integrated approach is necessary to deal with this disease. Services for the prevention of mother-to-child transmission of the human immunodeficiency virus (HIV) and other sexual and reproductive health initiatives ought to integrate syphilis elimination initiatives. The co-infections of HIV + HTLV-1 + *T. pallidum* were rarest in our study. Due to these low co-infections numbers, it was not possible to establish correlation between the diseases (Table 6). We found that social status had no impact on the occurrence of the common infections studied during pregnancy in Franceville.

There was a significant association between *T. gondii* seropositivity and increased age which can be attributed to the increased exposure to infection through the years (Table 3). The results of the current study highlight the need for awareness for toxoplasmosis as well as methods of prevention targeting not only pregnant women, but also women old enough to bear children.

The seronegative *T. gondii* prevalence (42,75%) found here was consistent with previous data (56%) from Franceville in 2007 [53]. Our results also seems to show a decline of seropositivity against *T. gondii* when compared to similar studies from 1995 and 1997 [54]. This decline may be explained by the recent improvements of hygiene in Franceville. Indeed, in industrialized countries, where hygiene is better, the prevalence of *T. gondii* is residual [55]. The high level of seronegative women against *T. gondii* and the high level of circulation of the parasite recently reported in Gabon [56] are disconcerting as these pregnant women run a higher risk of birthing babies with congenital toxoplasmosis. The *T. gondii* prevalence found here was consistent with the one reported in a study conducted in Lagos, Nigeria (60%) [57]. This similarity may be due to comparable climatic conditions, personal hygienic practices and socio-economic status in both countries.

We did not assess the immunoglobulin isotypes circulating in women to see if they expressed active *T. gondii* infection during their participation in the study. Previous data showed that IgM prevalence was stable between 1995 and 2007 (2.6%) [53].

Nearly 90% of the included pregnant women showed immunity against rubella suggesting a high transmission level of the rubella virus in Franceville. The prevalence of anti-rubella immunity decreased with age and seemed to be influenced by social status, since the students (and also the younger women) tended to show less immunity against this infection. This data is consistent with the increase in the number of contacts with the rubella virus according to age. Therefore, it could be considered an accumulative infection. A number of studies have attested to the presence of rubella immunity in different African countries such as Tanzania (2013), Benin (2013), Burkina Faso (2012), and Mozambique (2002) with a prevalence of 92.6%, 94% and 93.3% and 95.3%, respectively [51, 58–60]. Our results also show that a few pregnant women did not have either *T. gondii* or rubella antibodies which render them and their unborn children more vulnerable to these two infections.

In a study conducted in the Central African Republic from 2011 to 2012, 6% of pregnant women were found to be triple infected with HIV, *T. pallidum* and *T. gondii* [41]. In our study, this type of triple co-infection was not detected nor was it possible to link co-infections to any particular demographic factor.

## Conclusion

Toxoplasmosis, rubella, HIV, syphilis and HTLV-1 are frequent in varying degrees in pregnant women in Franceville. This evidence should obligate health officials to adopt systematic approaches for the detection and management of these infections that torment vulnerable populations such as pregnant women and their unborn children. Particular attention needs to be paid to younger women who are still students as this group seems to be most affected by these infections. Further studies are needed to improve access to diagnosis and treatment of these infections, particularly for pregnant women, which will contribute to better antenatal care in the country.

## Abbreviations

CHRABF: Centre Hospitalier Régional Amissa Bongo de Franceville
CIRMF: Centre International de Recherches Médicales de Franceville
ELFA: Fluorescent Enzyme-Linked Assay
ELISA: Enzyme-Linked Immunosorbent Assay
HASG: Hôpital de l’Amitié Sino-Gabonaise
HIV: Human Immunodeficiency Virus
HTLV1: Human T-cell LymphotropicVirus Type 1
RPR-II: Phosphorothionate, 2-butenoic acid-3-(diethoxyphosphinothioyl) Methyl Ester
*T. gondii*: *Toxoplasmagondii*
*T. pallidum*: *Treponema pallidum*
UPARAM: Unité de Parasitologie Médicale
USS: Université des Sciences de la Santé
VDRL/TPHA: *Treponema pallidum*Hemagglutination Assay/Venereal Disease Research Laboratory
